# Kidney invasion occurred 2 years following liver transplantation for hepatic alveolar echinococcosis: a case report

**DOI:** 10.1186/s12879-023-08788-7

**Published:** 2023-11-10

**Authors:** Qirui Hu, Simin Chen, Yichen Fan, Qian Lu, Manjun Deng, Haining Fan

**Affiliations:** 1 Department of Hepatopancreatobiliary Surgery, Affiliated Hospital of Qinghai University, Xining, Qinghai, 810001 China; 2 Qinghai Research Key Laboratory for Echinococcosis, Xining, Qinghai, 810001 China; 3 State Key Laboratory of Pathogenesis, Prevention and Treatment of High Incidence Diseases in Central Asia, Clinical Medicine Institute, Urumqi, 830054 China; 4 The First Affiliated Hospital of Xinjiang Medical University, Urumqi, 830054 China; 5 Department of Hepatopancreatobiliary Surgery, Tsinghua Changgung Hospital, Tsinghua University, Beijing, 102218 China

**Keywords:** Alveolar echinococcosis, Liver transplantation, Albendazole, Case report, Immunosuppression therapy

## Abstract

**Background:**

The organ most commonly invaded in echinococcosis is the liver; the lungs, brain, kidneys, heart, and spleen are rarely invaded, and multi-organ involvement in echinococcosis is even rarer. No studies have reported renal invasion after liver transplantation for hepatic alveolar echinococcosis.

**Case presentation:**

We report here a case of renal invasion 2 years after allogeneic liver transplantation in a 53-year-old female patient with hepatic alveolar echinococcosis combined with lung metastases. At the time of the first consultation, the lesion had been found to involve the second hepatic hilum combined with lung metastases, but the patient requested conservative treatment, and the lesion was not controlled by taking albendazole for 3 years. After discussion in the treatment group, it was decided to use allogeneic liver transplantation and lung segmental resection for surgical treatment, after which the patient was put on long-term oral immunosuppression. She was hospitalized 2 years later for low back pain and diagnosed with renal alveolar echinococcosis. Due to significant compression and left-sided renal insufficiency, the final option was to remove the diseased kidney. It is worth mentioning that signs of unexplained urinary tract infection were present throughout the course of treatment.

**Conclusion:**

This study suggests that extra attention should be paid to the presence of cryptogenic lesions in patients with hepatic alveolar echinococcosis who already have definite metastatic lesions. Immunosuppressive drugs after liver transplantation in patients with hepatic echinococcosis may cause occult lesions to develop into active ones. In clinical practice, particular attention should be paid to patients with hepatic alveolar echinococcosis with long-term concomitant signs of unexplained urinary tract infections, which may be a precursor clinical feature of cryptogenic renal alveolar echinococcosis.

Echinococcosis, a zoonotic disease caused by cestodes of the genus Echinococcus (family Taeniidae)。Echinococcosis refers principally to two severe zoonotic tapeworm diseases, cystic echinococcosis (CE) and alveolar echinococcosis (AE), caused by Echinococcus granulosus sensu lato and Echinococcus multilocularis, respectively [1]. The organ most commonly affected by echinococcosis is the liver, followed by the lungs, with brain, spleen, kidney and heart rarely involved [2]。At present, hepatectomy is considered to be the most effective and radical treatment, but for patients with huge lesions or invasion of major vessels (portal vein, hepatic vein, vena cava), radical treatment can only be achieved through liver transplantation.If left untreated, the mortality rate reaches 90% after 10 to 15 years [1, 3, 4]. We report a case of renal alveolar echinococcosis in a patient with hepatic alveolar echinococcosis with lung metastases who developed renal alveolar echinococcosis after treatment with liver transplantation and partial lung resection. We hope that the discussion of the diagnostic process and disease progression will serve as a reference and warning for future clinical management of similar cases.

## Case presentation

This case report is about a 53-year-old female patient, living in the Tibetan plateau, with a history of close contact with animals such as “cows, sheep and dogs”, who was admitted to the hospital in 2017 with a 1-week history of back pain and discomfort. The patient had not received any treatment prior to admission, and physical examination revealed only mild epigastric tenderness.Tests for tumor markers such as alpha-fetoprotein (AFP), CA125 and carcinoembryonic antigen (CEA) were all negative, and the results of the Echinococcus IgG antibody test (ELISA) were positive.Routine urinalysis showed a mild urinary tract infection (urinalysis combination: bacteria: 23065.50/uL, turbidity: +1), but urine culture results were negative.

**Fig. 1:**
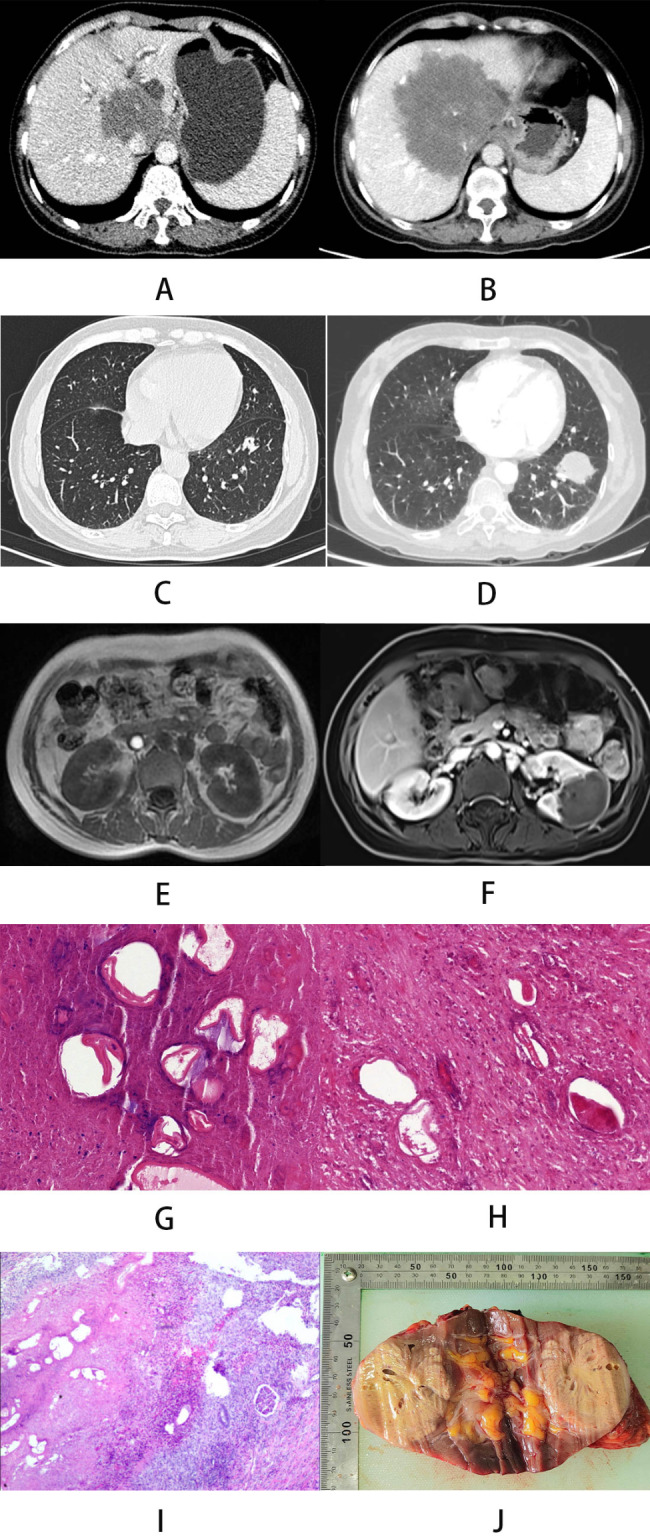
CT and MRI manifestations of the lesion and pathological examination and macroscopic view of the excised: (A) CT image of hepatic alveolar echinococcosis in 2017; (B) 2020 Liver lesions were more invasive than before; (C) CT images of lung lesions in 2017; (D) Enlargement of the left lung lesion in 2020; (E) MRI images of the kidneys did not show abnormalities in 2020; (F) MRI images suggestive of renal space-occupying lesions in 2020; (G) Pathologic examination of liver lesion location; (H) Pathologic examination of lung lesion location; (I) Pathologic examination of renal lesion location; (J) Macroscopic view of left renal lesion

An enhanced CT scan showed a lesion measuring approximately 5.83 cm x 5.1 cm with invasion of the second porta hepatis (Fig. 1A). Chest CT showed a microscopic nodular shadow (1.10 cm x 1.02 cm) in the lower region of the left lung (Fig. 1C), and no abnormalities were seen on nephrography. The diagnosis of hepatic alveolar echinococcosis was made based on his life history, CT scan findings and laboratory findings.

Subsequently, the patient herself refused to be treated with transplantation surgery and was treated conservatively with albendazole. Patients received 20 mg/kg/day (1280 mg/day) in 2 divided doses for 1 month, followed by continued treatment after 14 days of withdrawal. Back pain is relieved by conservative treatment. Three years later, he had sudden onset and worsening of back pain, and the degree of hepatic lesion invasion increased compared with the previous one (Fig. 1B), and the nodular shadow of the left lower lobe of the lung was enlarged (4.8 cm x 3.6 cm) (Fig. 1D).Features of urinary infection remained but renal imaging did not show any abnormality (Fig. 1E). Split allograft liver transplantation and left lower lung basal segmentectomy were selected for treatment after discussion by the treatment team, and postoperative pathology of liver lesions showed liver tissue structure was destroyed, irregular echinococcal cysts of varying sizes were seen consisting of alveolar structures with stratum corneum formation, and inflammatory cell remnants were seen in the surrounding, which was diagnosed as hepatic alveolar echinococcosis (Fig. 1G), and the pathology of lung lesions showed lung tissue structure was destroyed, irregular echinococcal cysts of varying sizes were seen consisting of alveolar structures with a thinner visible cuticle formation, and the germinal layer was not easily recognizable. The diagnosis of alveolar echinococcosis was made (Fig. 1H).Long-term oral tacrolimus, but not albendazole, were administered after discharge. Two years after liver transplantation, the patient developed left-sided lumbar pain, and magnetic resonance imaging showed the presence of a space-occupying lesion approximately 3.80 × 5.06 cm in size in the left kidney (Fig. 1F), with obvious compression symptoms and renal dynamic imaging showed left-sided renal insufficiency. Elective resection of the left kidney was performed (Fig. 1J) and postoperative pathology showed glomerular atrophy and irregularly sized alveolar composed of echinococcal larval sacs with a thin stratum corneum surrounded by bands of acute and chronic inflammatory cell infiltration interspersed with eosinophils. Diagnosis of renal alveolar echinococcosis was made (Fig. 1I).

## Discussion

The liver is the primary target organ for Echinococcus multilocularis, which can also metastasize to the lungs, brain, kidneys, heart, and spleen. Gross anatomic features usually consist of a large or single nodular, yellow or white cystic mass with a firm texture [1]. The history of this patient is highly complex; the patient underwent allogeneic liver transplantation and segmental lung resection for hepatic alveolar echinococcosis combined with lung metastases, and 2 years later developed renal alveolar echinococcosis and underwent nephrectomy due to severe compression and left-sided renal insufficiency; no similar case report has been found previously.

Dealing with the imaging-positive lesion was carried out throughout the treatment protocol, and since we were unable to achieve radical resection by partial hepatectomy due to the invasion of the lesion into the second hepatic hilum, split allograft liver transplantation and left lower lung basal segmentectomy was the most appropriate treatment option for this patient, who at this point had had a complete resection of the imaging-positive lesion. No renal imaging prior to immunosuppression revealed any abnormalities, but the development of renal alveolar echinococcosis 2 years later was unexpected. In this regard, we prefer the conclusion that persistent occult renal infections develop into active lesions after the administration of immunosuppressants [1, 4–8]. It is still believed that the liver is the first infected organ in secondary infections, but the transplanted liver at the time of nephrectomy had no lesions on imaging. On the other hand, according to the follow-up results, this patient relocated away from the infected area during his conservative treatment with albendazole after the initial diagnosis. In summary, the possibility of re-infection was basically excluded. In addition, signs of unexplained urinary tract infection persisted throughout the course of treatment. Bentani N,Yousofi DH et al. found that renal echinococcosis is usually secondary to disseminated echinococcosis and is accompanied by the clinical features of a prolonged urinary tract infection [9–11]. Although it is not known whether occult infections have this feature, in the context of this case it is reasonable to suspect that occult infections may also have this feature.

Management of imaging-positive lesions is currently the mainstay of echinococcosis treatment, which undeniably increases the likelihood that occult infections will be missed. We hope that this case report will alert clinicians that, firstly, patients with clear metastatic lesions should be given extra attention for the presence of occult lesions. Secondly, because liver transplant recipients take immunosuppressive drugs after surgery, this may cause occult lesions to develop into active lesions.

## Conclusion

Imaging-negative cryptogenic renal alveolar echinococcosis may also present with clinical features of urinary tract infection. Particular attention should be paid in clinical practice to patients with hepatic alveolar echinococcosis with long-standing concomitant signs of unexplained urinary tract infection, which may be a precursor clinical feature of cryptogenic renal echinococcosis.

## Data Availability

All data generated or analysed during this study are included in this published article.
